# Towards a computer aided diagnosis system dedicated to virtual microscopy based on stereology sampling and diffusion maps

**DOI:** 10.1186/1746-1596-6-S1-S3

**Published:** 2011-03-30

**Authors:** Philippe Belhomme, Myriam Oger, Jean-Jaques Michels, Benoit Plancoulaine, Paulette Herlin

**Affiliations:** 1 GRECAN EA 1772, IFR ICORE 146, Université de Caen, France

## Abstract

An original strategy is presented, combining stereological sampling methods based on test grids and data reduction methods based on diffusion maps, in order to build a knowledge image database with no bias introduced by a subjective choice of exploration areas. The practical application of the exposed methodology concerns virtual slides of breast tumors.

## Introduction

While pathologist population tends to dramatically dropped, the number of pathological cases to examine increases steadily (mainly due to the new screening campaigns). Fully automated image processing is able to provide a solution to this problem. Indeed, it may help pathologists in their daily practice in finding objective criteria for differential diagnosis or quantifying prognostic markers.

The recent marketing of digitizers now allows visualizing the entire histological slide at high resolution, while limiting time expense and artifacts previously encountered with image tiling methods [1]. More and more introduced in pathology departments, these systems however generate very large images which frequently exceed several Gigabytes. Because of tumor heterogeneity, it is essential to build image knowledge databases containing representative features of the various morphological types of lesions before considering implementing computer-aided diagnosis systems [2]. But, as it is almost impossible for a pathologist to manually segment such a large image, and a fortiori many of them (the estimated time being hundred hours), the current practice consists in manually selecting some 'representative areas'. A bias is then introduced in the process as this choice is obviously subjective. It is then mandatory to find wiser solutions leading to an unbiased collection of image databases. The sampling tools offered by stereology can be of great help in this context [3,4].

Systematic sampling resulting from a random starting point with a fixed periodic interval is able to reduce the area to be analyzed, while preserving the collection of varied and characteristic regions encountered in a virtual slide (VS) of a tumor. However, even if the working area is smaller, the number of selected regions can be very high and can include many redundant elements. A data reduction has then to be conducted in order to keep a proper right number of representative elements. Among these reduction methods, the diffusion maps [5,6] provide a very attractive framework for processing and visualizing huge non-linear bulk data.

This work relates to the medical image processing and retrieval field, with the goal to develop and propose a functional computer-aided diagnosis system based on a knowledge database. The original strategy exposed in this paper consists in starting from a collection of VS, then taking advantage of stereological sampling methods and diffusion maps, to finally compute a knowledge image database containing a small number of image patches that are representative of a given histological type or subtype. The practical application illustrating this framework makes use of VS of breast tumors.

## Materials

Images used for illustrating the strategy are VS of histological sections of breast tumors, stained in the same laboratory according to the Hematoxylin-Eosin-Safron protocol and acquired with the same digital scanner. The main goal here is to collect a useful number of image patches corresponding to a given histological type. Its ability to be embedded into a computer-aided diagnosis system (CADS) is illustrated by building an unbiased image database containing representative patches of a benign tumor (Fibroadenoma) and by testing the discrimination between a benign tumor and a malignant tumor (Fibroadenoma vs Comedo carcinoma). Images have been acquired at X20 (0.5 µm per pixel), using a digital slide scanner (ScanScope CS from Aperio Technologies, Inc) and then stored in TIFF 6.0 image file format with a 30% jpeg compression [7]. Their mean size is about 65000x43000 pixels^2^ and each holds about 350 MB on a hard disk.

The tools needed for this study were developed in Python language (http://www.python.org) with the help of specialized modules (PIL: Python Imaging Library and SciPy: http://www.scipy.org).

## Methods

### Stereology

In order to reduce the expertise workload, a stereological test grid for point counting is over imposed onto VS in ImageScope viewer (Aperio Technologies, Inc) [8]. This kind of probe is usually dedicated to estimate the area and volume fractions in a tissue compartment [4]. In our application, the grid step was set to 1000x1000 pixels, ie 2800 points in an image having an average size of 65000x43000. The pathologist has to determine in which histological class must be arranged each area centered on grid points; 30 possibilities are available (breast tumor histological types and sub-types) provided by the annotation tool embedded in Aperio ImageScope. The pathologist is only asked to draw on each point a simple line selected in the overlay layer whose name corresponds to his choice. Each area is then extracted by a dedicated software at the plain resolution and stored as an uncompressed TIFF image file in order to enrich the future knowledge database. These areas (called later 'patches') are squares of size 400x400 pixels. Patch size has been chosen according to the mean size of representative structures encountered in the various histological types of breast tumors [9]. It allows pathologists to expertise only 16% of the whole VS. All patches are then analyzed and sorted in order to storing only the most representative ones. The original image name, the histological type of the patch and its coordinates in the test grid are stored in each filename, for later being used by sending SQL requests to the database.

### Patch characterization

For each patch, statistical features are computed and embedded in a vector signature. All these signatures will be used in a later image retrieval process. At this stage of the study, none of the features results from segmentation. All are obtained from global measurements on patches computed on *I*_1_*I*_2_*I*_3_ and *YCh*_1_*Ch*_2_ color components which are derived from the *RGB* color system according to the following formulas proposed by Ohta [10] and Carron [11]:.

These color components have been computed from the *RGB* histograms previously reduced to 64 values.

For a given color component whose histogram is called *H*, the computed features are: *H*, *H* reverse sorted, cumulative *H*, 20%, 40%, 60% and 80% quantiles of cumulative *H*, meanH, medianH, modeH, SkewnessH, KurtosisH, PearsonModeSkewnessH, that is a total of 13 data. Three of them are themselves vectors of 64 values, but will provide a single feature after distance measurements between two signatures. Definitions of these statistical features can be found in [12]. With the resulting 5 effective color components (as *Y*=*I*_1_), 65 distance measures will be taken into account but 1010 values will be stored in the signature vector for each patch. Considering the sparse numerical range of features in signatures, the Kullback-Leibler symmetrical distance has been retained for its ability to manage such values, while remaining simple and fast to implement (compared to Mahalanobis or earth mover's distance for example). The symmetric Kullback-Leibler distance between two vectors *p*_1_,*p*_2_ of length n is defined by:.

The computation time can be reduced using:.

In order to give the same weights to histogram features (*h_i_*) and scalar features (*x_i_*), the Kullback-Leibler distance is averaged by the number of histogram values while comparing *h_1_* and *h_2_*. Because of the symmetry of *D_KL_*, and with *N* images to process, the computation time is proportional to  and is parallelized on multi-core/multi-processor computers.

### Data reduction

The ultimate goal of this study is to contribute to the development of a computer-aided diagnosis system (CADS) whose one component should be a visualization tool dedicated to knowledge image databases. This tool would be useful for pathologists if results can be visualized in a 2D or possibly 3D space. It is therefore necessary to reduce dimensionality from n (65 dimensions in our example) to 2 or 3. The patches signatures do not necessary contain linear data. Therefore it is not appropriate to perform a principal component analysis (PCA). Belkin [5] and Coifman [6] have shown that methods based on diffusion maps, involving eigenvalues and eigenvectors of a normalized graph Laplacian, are well suited to non linear data.

Let *X*={*x*_1_,*x*_2_,...,*x_N_*} be a set of *N* patches. A (*N*x*N*) kernel *P* is obtained whose coefficients are:

 where  and .

The eigenvectors φ_k_ of *P*, ordered by decreasing eigenvalues, give the axes of the new observation space. It must be noticed that φ_0_ is never used since linked to the eigenvalue λ=1 (ie the mean of the data set). The projection is then done in (φ_1_, φ_2_) for a 2D space or (φ_1_, φ_2_, φ_3_) for 3D. The choice of ε is empirical but should permit a moderate decrease of the exponential; the median of *D_KL_* distances is usually chosen [13].

## Results

**Figure 1:**
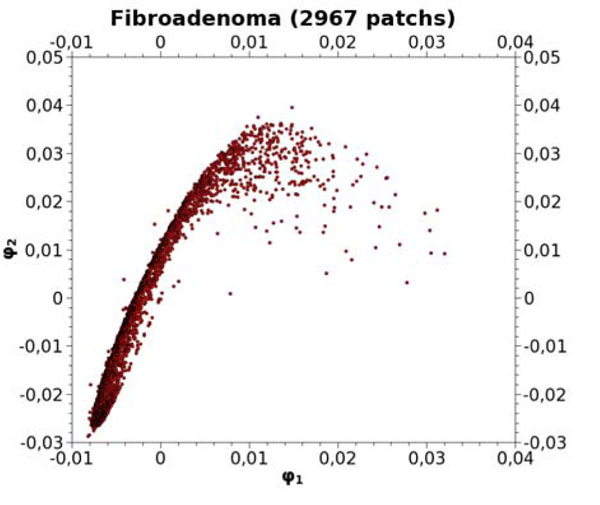
2D projection of Fibroadenoma patches

To illustrate the data reduction algorithm, 4 VS coming from different pathological cases have been selected; their storage needs 1,5 GB. A total number of 2967 patches, classified as Fibroadenoma by a pathologist, have been extracted from a stereological test grid. Figure 1 shows their projection in a 2D space. In this reduced space, a classical Euclidean distance can be applied to estimate the similarity between two different patches.

**Figure 2:**
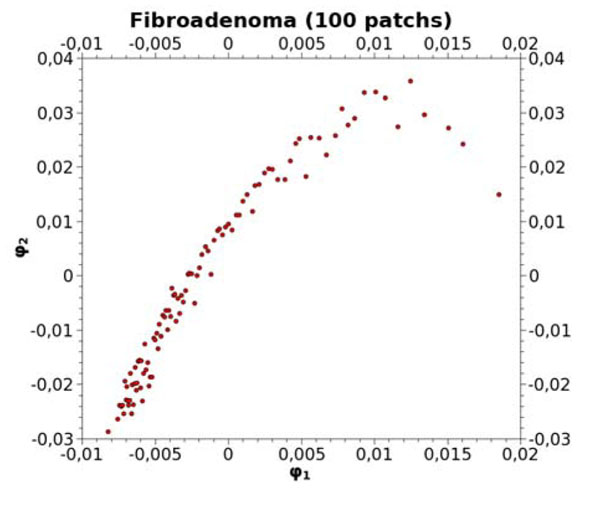
Selected Fibroadenoma patches

Keeping only 100 representative elements thanks to a regular decimation along the original curve, one obtains a new set of patches to be stored in the knowledge database. Their 2D projection is illustrated in Figure 2. These patches represent the unbiased reference, to which new 400x400 areas, extracted from unknown VS, should be compared.

**Figure 3:**
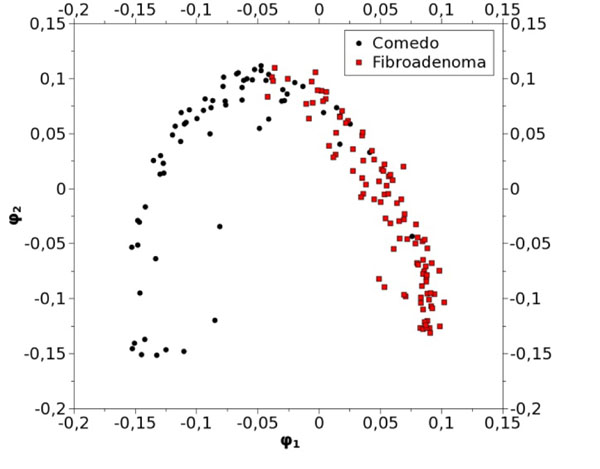
2D projection of Fibroadenoma vs Comedo carcinoma (164 patches)

To illustrate the comparison between a benign and a malignant tumor, these 100 patches obtained from the Fibroadenoma class, were compared to 64 patches extracted from a Comedo carcinoma. Figure 3 exhibits the 2D projection of the overall 164 patches. The good discrimination of these two families is obvious, especially according to the axis φ_1_=0 which usually provides the sharpest separation between object classes [13].

## Conclusion

This work relies on an original strategy starting from VS and leading to an unbiased knowledge image database containing reference patches of breast tumors. We have shown that combining stereological sampling and data reduction based on diffusion maps offers an interesting general framework for this purpose. Once the sequence of procedures has been implemented, the only parameters to be tuned are the choice of image features to use for patch signatures, the size of patches and their number to be kept in the knowledge database. At this stage of the study, none of the features come from any segmentation or from any texture measurement. When all steps will be validated, it will be time to consider advantages of adding new parameters or to introduce other color components. Using patches acquired at a lower resolution should be also an interesting issue, depending on the histological type or subtype to be studied. It should be noticed that, up to now, we did not try to adjust parameters in order to be independent from the acquisition conditions, since all images come from the same origin. However, the final goal being the development of a CADS for several laboratories, it should be necessary to take this into account, by computing International Color Consortium profiles for each device used along the process, starting from histological staining up to image acquisition [14].

The results illustrated in this study are preliminary ones. Up to now, 400 high resolution VS of breast tumors are available. The benign and malignant tumors were classified into 30 histological types and subtypes. We plan now to project these 30 classes in the same 3D space, in order to analyze their scattering. This work is in progress in our laboratory.

## Competing interests

The authors declare that they have no competing interests.
